# Expression of Metazoan Annexins in Yeast Provides Protection Against Deleterious Effects of the Biofuel Isobutanol

**DOI:** 10.1038/s41598-019-55169-9

**Published:** 2019-12-09

**Authors:** Carl E. Creutz

**Affiliations:** Department of Pharmacology, University of Virginia, Charlottesville, VA 22908 USA

**Keywords:** Membrane trafficking, Industrial microbiology, Applied microbiology, Environmental impact, Membrane biophysics

## Abstract

The ability of microorganisms to produce biofuels by fermentation is adversely affected by the perturbing effects of the hydrophobic biofuel on plasma membrane structure. It is demonstrated here that heterologous expression of metazoan, calcium-dependent, membrane-binding proteins of the annexin class can reduce deleterious effects of isobutanol on *Saccharomyces cerevisiae* viability and complex membrane functions. Therefore, expression of annexins in industrial strains of yeast or bacteria may prove beneficial in biofuel production.

## Introduction

Due to the environmental effects of the generation of carbon dioxide from the burning of fossil fuels, and to the limits on the availability of oil, coal and natural gas, there is interest in the production of synthetic biofuels by fermentation of renewable organic materials^1–3^. A successful example is the production of ethanol as a substitute for gasoline at the level of 10 to 15% in automobile engines. Current research and development in this area is shifting toward the production of intermediate chain alcohols such as isobutanol because these are a better substitute for gasoline in current technologies. However, since isobutanol and other alternatives are more hydrophobic than ethanol, they are more toxic to the microorganisms designed to produce the compounds because they disrupt the structure of the lipid bilayer forming the core of the cell membrane^4,5^. Presented here is a novel method to potentially by-pass this limitation of current technology: the creation of synthetic hybrid microorganisms that express proteins from higher organisms that stabilize the membranes of the microorganisms against the disruptive effects of the hydrophobic compounds. This is accomplished by transforming the microorganisms with genes that express calcium-dependent, membrane-binding proteins of the annexin class^6^.

In biophysical studies the annexins have been shown to stabilize synthetic model membranes *in vitro* against disruption by a wide range of hydrophobic or amphipathic compounds, and to promote their repair after disruption^7^. The results described in this report provide evidence that a similar membrane stabilizing effect can apparently be achieved in a living cell by expressing these proteins in a microorganism, *Saccharomyces cerevisiae*, that can be used for biofuel production.

**Figure 1:**
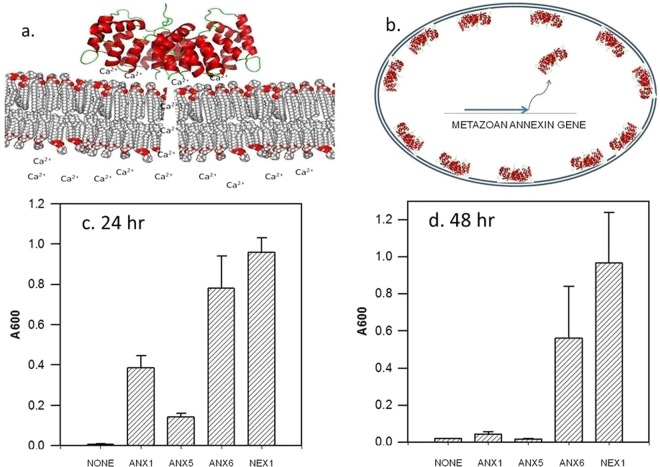
Expression of annexins enhances the survival of yeast exposed to 2% isobutanol. (**a**) Schematic illustration of the role of annexins in blocking membrane damage caused by isobutanol. Isobutanol disrupts the packing of phospholipids in the cell membrane bilayer, allowing calcium to flow through the membrane into the cell interior. The annexin binds to the site of calcium entry due to the ability to bind phospholipid headgroups coupled with the binding of calcium. This leads to the reorganization and packing of the lipids, repairing the membrane damage. The actual molecular structures of the permeability pathway and the repaired membrane are not known. (**b**) Annexins are expressed in yeast from transfected metazoan annexin genes providing protection to the microbial cell membrane. (**c,d**) Viability of yeast cultures grown in 2% isobutanol for 24 (**c**) or 48 (**d**) hours is enhanced by expression of annexins (NONE, empty expression vector; ANX1, human annexin A1; ANX5, human annexin A5; ANX6, human annexin A6; NEX1, *C. elegans* Nex-1 annexin). Viability was determined by dilution of the isobutanol-treated cultures into normal medium and measuring the A600 of the cultures after overnight growth. Means +/− s.d. (n = 3) are plotted. P values (student’s two tailed t test versus empty vector) are for 24 hours (**c**) ANX1 0.00037, ANX5 0.00027, ANX6 0.0011, Nex1 0.000024; for 48 hours (**d**) ANX1 0.0217, ANX5 0.0341, ANX6 0.0293, Nex1 0.0039. P values for differences between the individual annexins are given in Supplementary Table S1.

Studies of the interaction of annexins with model membranes suggest a possible mechanism by which annexins could protect cell membranes *in vivo*^7^. The annexins bind to the headgroups of negatively charged phospholipids on membrane surfaces, but do so only in the presence of high concentrations of the calcium ion, Ca^2+^. Inside cells the concentration of calcium is normally maintained at a level too low to promote this interaction. Therefore, an annexin can be expressed in a microbial cell in a relatively inert state, one that does not interfere with normal cellular processes. However, if the cell membrane is disrupted, the normal permeability barrier to calcium is lost and calcium will enter the cell and cause the annexin to bind to the membrane specifically at the point where the calcium level has become high because of damage to the membrane structure. By binding to the lipid headgroups the annexin stabilizes the membrane structure, restoring the normal permeability barrier of the membrane. The annexin may thus act as a “molecular bandage” targeted specifically to the sites of membrane wounding (Fig. 1a,b).

## Results

cDNAs for human annexins A1 (ANX1) A5 (ANX5), and A6 (ANX5), and for the Nex-1 annexin (NEX1)^8^ from the nematode, *Caenorhabditis elegans*, were expressed in yeast cells using a yeast/E. coli shuttle plasmid with a *LEU2* gene for selection in a *LEU2*^−^ auxotrophic yeast strain^9^. The expression of the annexins was under control of the *GAL10* promoter and cultures were grown in 2% galactose in defined medium to activate the *GAL10* promoter. Control experiments were performed with the empty plasmid vector, or by substituting 2% glucose for the 2% galactose in order to repress the *GAL10* promoter.

The ability of the annexins to reduce the deleterious effects of isobutanol on yeast cells was determined in two types of experiments. First, yeast cultures were inoculated into growth media containing a concentration of isobutanol (2%) that completely blocked cell growth, as monitored by measuring the optical density of the cultures at 600 nm over time and by examination by light microscopy. After incubation in this medium for 24 hours or 48 hours, the viability of the yeast cells was determined by diluting the cultures into normal medium and monitoring the subsequent increase in optical density. As shown in Fig. 1c,d the expression of the annexins allowed the yeast to survive in a concentration of the alcohol that is lethal to the yeast in the absence of the annexins. In contrast, control cultures grown in glucose medium to repress the *GAL10* promoter were not viable after 24 hours of incubation in 2% isobutanol.

**Figure 2:**
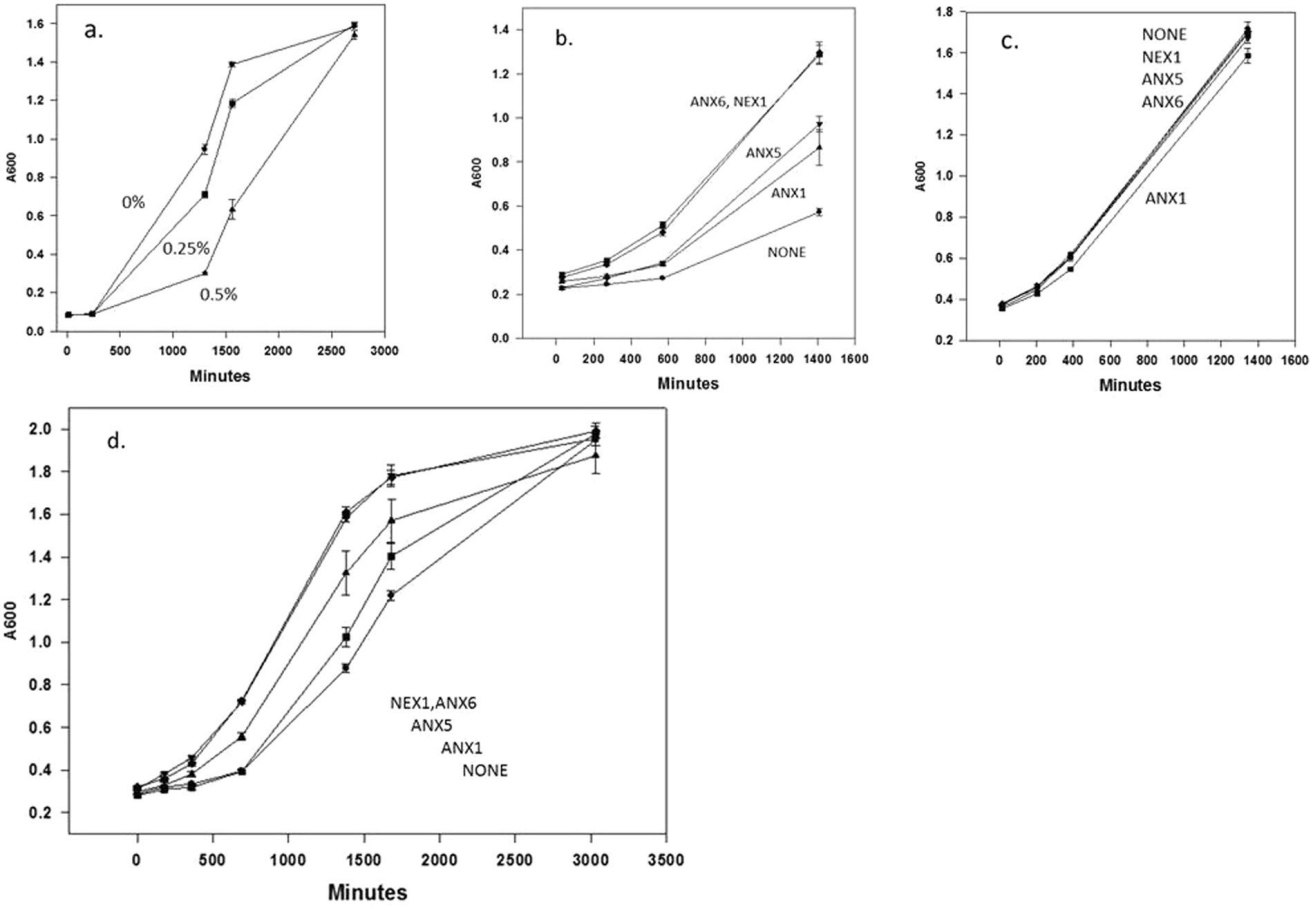
Expression of annexins restores the ability of yeast in 1% isobutanol to adapt rapidly to growth in galactose. (**a**) Transfer of control yeast, transformed with the empty expression vector, from a glucose containing medium to a galactose containing medium is associated with a delay in growth while cells adapt to the change in sugar. As shown, the delay is increased by the addition of 0.25% or 0.5% isobutanol. (**b**) Expression of annexins enhances the initial growth rate of yeast diluted from glucose-containing medium into galactose medium containing 1% isobutanol. The data are means +/− s.d. (n = 3). P values (student’s two tailed t test versus empty vector) at 1400 minutes are ANX1 0.0036, ANX5 0.00007, ANX6 0.000015, Nex1 0.000013. P values for differences between the individual annexins are given in Supplementary Table S2. (**c**) Expression of the annexins does not alter the initial growth kinetics when the yeast cells are diluted from galactose-containing medium to galactose containing medium containing 1% isobutanol. (**d**) After the switch from glucose to galactose, the full growth curves in 1% isobutanol suggest that annexin expression does not influence subsequent log phase growth or culture density at saturation. Due to the differences in the lag phase, the cultures have different densities at intermediate points. The data are means +/− s.d. (n = 3). P values (student’s two tailed t test versus empty vector) at 1380 minutes are ANX1 0.0075, ANX5 0.0018, ANX6 0.000010, Nex1 0.000012. P values for differences between the individual annexins are given in Supplementary Table S2.

The second type of experiment revealed an ability of the annexins to protect a complex membrane-remodeling event: adaptation of the yeast cell membrane that occurs during a shift from growth in glucose to galactose as a carbon source. This adaptation includes the insertion of galactose transport proteins into the cell membrane, a process involving exocytotic fusion of galactose permease-containing membrane vesicles with the plasma membrane^10^. This adaptation process was significantly retarded by the presence of 0.25 or 0.5% isobutanol (Fig. 2a). In the presence of 1% isobutanol and 2% galactose, expression of the annexins enhanced the initial rate of growth of yeast 3 to 4 fold after dilution from a saturated culture initially grown in 2% glucose (Fig. 2b). However this only occurred if the preculture was grown in glucose medium and not in galactose medium before diluting into galactose medium (compare with Fig. 2c); presumably the precultures grown in galactose were already adapted to galactose.

Analysis of the full growth curves to saturation indicated that the beneficial effect of the annexins was a reduction in the lag time in the growth curves as the yeast were adapting to the change to galactose medium (Fig. 2d). The growth curve in the absence of annexin expression reached half-saturation at 1700 minutes, while expression of annexin A6 or the Nex-1 annexin led to half-saturation at 900 minutes. This represents a reduction of the lag time by 800 minutes (13.3 hours). The exponential phase of growth after adaptation to galactose, and the final cell density at saturation were not altered by expression of the annexins. In contrast, in the absence of isobutanol, the expression of the annexins did not alter the lag time for adaptation to galactose (confirmed in this study, as well as previously reported^9^). The degree of reduction of the lag time was different for the different annexins, and the order of the effectiveness of the annexins in this assay was similar to the order of effectiveness of the annexins in protecting viability in the higher concentration (2%) of isobutanol: Anx6 ~ Nex1 ≫ Anx1 ~ Anx5. This suggests the mechanism of action of the annexins in both assays may be related.

## Discussion

### Membrane repair by annexins: “macroscopic” and “microscopic” repair mechanisms

Evidence has previously been obtained from a number of experimental models that annexins can participate in the repair of damaged animal cell membranes^11–17^. These include diverse models of membrane rupture such as osmotic lysis, mechanical damage with micropipets, laser perforation of cell membranes, and membrane lytic toxins. In these models the annexins move to the sites of membrane damage presumably as a result of interactions with the membranes stimulated by the entry of calcium at the site of membrane wounding. In many of these models there is additional evidence that other proteins supplemental to the annexins might participate in the repair process, such as synaptotagmin^18,19^ or dysferlin^13^, and complex membrane remodeling, such as the insertion of membrane patches or exocytic vesicles could occur to provide additional membrane surface in the repair. Knockdown or deletion of annexins in these models tends to inhibit the repair process, demonstrating the importance of the annexins in the repair process. Repairs to such major sites of membrane damage may be thought of as “macroscopic” membrane repair.

In contrast, annexins may also repair minor disruptions in membrane permeability caused by small, amphiphilic or charged molecules, as may be occurring in the repair of yeast membranes permeabilized by isobutanol in this study, or in model systems with pure liposomal membranes^7^. In these cases it appears the annexins can act alone to reorganize and stabilize the lipid components of the membrane to restore the impermeability barrier to ions, metabolites, and possibly protein molecules. This type of repair by annexins might be termed “microscopic” membrane repair as opposed to the macroscopic repair mechanisms described above. However, it also seems likely that microscopic and macroscopic pathways for repair might proceed in concert in cells. For example, the insertion of a large membrane patch or vesicle to fill in a large hole in a membrane might be a leaky process, and the leakage could be suppressed by the action of annexins to promote a final seal of the membrane patch. In a similar way annexins may have a general role in the cell as “leak protectors” for a number of dynamic membrane processes.

### Differential efficacies of the annexins in yeast membrane protection

Although the same *GAL10* promoter was used to express the different annexins in yeast in this study, the annexins exhibited differential efficacies in suppressing the damaging effects of isobutanol. This might have been due to different levels of annexins due to differential stability of the annexins in yeast. However, in previous studies of the yield of annexins isolated from similar yeast expression systems^9,20^, the order of yields was ANX6~ANX5 > ANX1 > NEX1 (0.216, 0.213, 0.148, 0.070 mg protein/gm of cells, respectively). This does not correlate with the relative degrees of effectiveness of the annexins in suppressing the effects of isobutanol seen here (ANX6 ~ NEX1 ≫ ANX1 ~ ANX5).

The differential efficacies of the annexins may reflect intrinsic abilities of the annexins to restore or protect membrane order. This would likely be influenced by the affinity for calcium and/or affinity for specific yeast membrane lipids. In *in vitro* studies with defined lipids it is found that different annexins have different affinities for different phospholipids, or mixtures of phospholipids, and their calcium dependence of binding to lipids also depends on the specific lipid headgroups present^6,21–26^. For example, annexin A5 has a high affinity for calcium in the presence of mammalian lipids^6^ while the Nex-1 annexin has a low affinity for calcium in the presence of mammalian lipids^20^. Nonetheless, the Nex-1 annexin was more effective at protecting yeast membranes than was annexin A5 in these experiments. However, there is very little data available on the characterization of annexin interactions with endogenous yeast lipids so it is not clear if differential calcium or lipid affinities explain the differences in membrane protection seen here.

It is also possible that the different annexins may localize to different parts of the yeast cells that are of different importance in protecting against membrane damage. However, the intracellular distributions of the mammalian annexins in yeast have not been studied. In the long run, all of these factors described above may prove to be important to consider in optimizing the technology described here to enhance yeast function in biofuel production.

### Role of annexins in protecting membrane trafficking events in isobutanol-treated yeast

As demonstrated in this study, isobutanol retards the ability of yeast to adapt to the shift from growth in glucose to growth in galactose, and the annexins are effective at suppressing this effect of isobutanol. The adaptation process requires the insertion of galactose transporters into the cell membrane, a process that occurs by fusion of transporter-containing vesicles into the cell membrane. It is not clear how the annexins provide protection for this event. They may act to protect the membrane structure necessary for the exocytotic event to occur, or they may directly promote the membrane fusion event per se, or possibly act through both mechanisms. Additional evidence that the annexins may be beneficial in promoting this process in yeast comes from a previous study on the effects of annexins on the yeast secretory (*sec*) mutants. Annexins were found to reduce the lag time for adaption to a shift from glucose to galactose in the case of yeast with a temperature sensitive mutation in the *SEC2* gene^9^. This gene encodes a GTPase-activating protein for the small GTPase Sec4p involved in exocytosis. The insertion of the galactose permease into the plasma membrane by exocytosis is compromised by defects in the *SEC2* gene^27^, and this was partially corrected by heterologous expression of annexins^9^. It is possible that isobutanol in the current experiments had a similar effect on membrane structure and function as the *sec2* mutation. However, some differences were seen in the effectiveness of the annexins in reducing the lag time in these differently compromised systems. In the *sec2* mutants annexin A1 was slightly more effective than annexin A6 in correcting the deficit in the lag time^9^, while in the isobutanol-treated yeast annexin A6 was more effective than annexin A1. Since the Sec2p protein must interact with other late Sec proteins to accomplish its role in exocytosis, it may be that isobutanol alters the function of several late Sec proteins whereas the *sec2* mutation would only directly affect the Sec2p protein itself. This difference in mechanism might account for the different relative effectiveness of the annexins in two situations.

### Implications for biotechnology applications of the annexins

The beneficial effects of annexin expression on membrane trafficking and cell viability compromised by isobutanol might translate into better efficiencies of biofuel production, although this has not been directly tested. Preliminary tests conducted during this study with butanol, isoamyl alcohol, and hexanol indicated that annexin expression is also beneficial in the case of these alcohols, suggesting the technology may have broad applications for the production of biofuels and other hydrophobic or amphipathic compounds, including pharmaceuticals.

Metazoan annexins have frequently been expressed from plasmids in *E. coli*, and, when isolated from these expression systems, the annexins have characteristic membrane-binding activities seen with the native proteins isolated from animals^28–34^. Therefore, the membrane-protecting properties of the annexins might also be manifest in prokaryotic cells used to produce useful compounds from more complex substrates such as crop wastes amenable to bacterial digestion^3^.

## Methods

### Annexin expression vectors and yeast strain

The annexin expression vectors were constructed as previously described^9,20,35^ by inserting annexin cDNAs into vector YEpC^2^1 or YEpDB60 which incorporate the *GAL10* promoter and the *LEU2* auxotrophic marker for selection in leu^-^ drop-out media supplemented with 2 mM CaCl_2_ and containing 2% glucose or 2% galactose^36^. Expression of the annexins was verified by isolation and purification from culture extracts and demonstration of canonical calcium-dependent, phospholipid-binding of the isolated proteins^9,20,35^.

The host yeast strain NY606 (*MATα, GAL*^+^, *leu2-3,112*) was received from Peter Novick^9^.

### Monitoring of yeast cell growth in the presence of isobutanol

Yeast precultures were grown at 30 °C to saturation (2 to 4 days) in leu^−^ drop-out medium supplemented with 2 mM CaCl_2_ and containing 2% glucose or galactose. 5 ml cultures containing 0 to 2% isobutanol in 1.3 cm diameter cell culture tubes were inoculated with 200 ul of the precultures. The isobutanol was added to the medium and the mixture vortexed prior to adding the yeast cells. The cultures were then incubated on a rotator at 30 °C for 1 to 3 days.

Cells were grown in cell culture tubes with loose caps (“aerobic”), or with sealed caps (“anaerobic”). Aerobic cultures grew slightly more quickly than anerobic cultures, but the effects of the annexins and isobutanol on growth kinetics were similar in either case. To check for loss of isobutanol in the aerobic cultures, a mock experiment was performed with 2% isobutanol in water and the loss of isobutanol over 48 hours was checked by measurement of the optical density at 220 nm. No loss of isobutanol was seen. Due to the low absorbance of isobutanol, a loss of less than 10% of the isobutanol would not have been detected with the spectrophotometer used (Beckmann DU70). We conclude that loss of isobutanol during the experiments was less than 10% of total.

Optical densities at 600 nm of the cultures were measured by removal of samples from the cultures and measurement in a 1 cm path length quartz cuvette on a Beckmann DU70 spectrophotometer, or by measurement of the culture density directly in the 1.3 cm clear cell culture tubes on a Fisher Scientific Genesys 20 spectrophotometer.

Cell cultures were examined for contamination and morphology by light microscopy at the beginning, at intermediate time points, and at the end of the time courses. Under some conditions some morphological changes (elongated cells) were seen in the presence of isobutanol as reported previously for the effects of “fusel” alcohols^37^. There appeared to be some qualitative suppression of this morphological change with the expression of the annexins, but this effect was variable and was not quantified.

### Statistics

All experiments reported were repeated at least three times with independent isolates of the original yeast stocks. For each independent experiment triplicate cultures were prepared from a single preculture. Absorbances reported are means +/− s.d. (n = 3) and P values were determined using a two tailed student’s t test.

## Supplementary information

Supplementary Information

## Data Availability

Data and unique materials described here are available from the author upon reasonable request.
