# Supplementary material for: Expression of Metazoan Annexins in Yeast Provides Protection Against Deleterious Effects of the Biofuel Isobutanol
Source: Sci Rep. 2019 Dec 9;9:18603. doi: 10.1038/s41598-019-55169-9 (PMC6901584; doi:10.1038/s41598-019-55169-9)
Supplement: Supplementary file 1 — Supplementary Information [file 41598_2019_55169_MOESM1_ESM.pdf]

SUPPLEMENTARY INFORMATION

EXPRESSION OF METAZOAN ANNEXINS IN YEAST PROVIDES PROTECTION AGAINST DELETERIOUS EFFECTS OF THE BIOFUEL ISOBUTANOL

Carl E. Creutz, Department of Pharmacology, University of Virginia, Charlottesville, VA 22908

Supplementary Table S1: Statistics for Figures 1c and 1d. Means and standard deviations (n=3) for the A600 of control (NONE) and each annexin are given. P values are given for comparing the means for the annexins in the column on the left with the annexins listed in the headings (student's two tailed t test).

Figure 1c 24 hours

Annexin	mean	s.d.	Anx1 (P)	Anx5 (P)	Anx6 (P)	Nex1 (P)
None	0.0073	0.0012	0.00037	0.00027	0.0011	0.000024
Anx1	0.3873	0.0590		0.0024	0.0162	0.00047
Anx5	0.1423	0.0194			0.0024	0.000050
Anx6	0.7803	0.1592				0.155
Nex1	0.9580	0.0737				

Figure 1d 48 hours

Annexin	mean	s.d.	Anx1 (P)	Anx5 (P)	Anx6 (P)	Nex1 (P)
None	0.0210	0.0010	0.0217	0.0341	0.0293	0.00385
Anx1	0.0443	0.0110		0.0142	0.0336	0.00424
Anx5	0.0177	0.0015			0.0288	0.00381
Anx6	0.5603	0.2811				0.1464
Nex1	0.9670	0.2726				

Supplementary Table S2: Statistics for Figure 2b and 2d. Means and standard deviations (n=3) for the A600 of control (NONE) and each annexin are given. P values are given for comparing the means for the annexins in the column on the left with the annexins listed in the headings (student's two tailed t test).

Figure 2b A600 at 1400 min

Annexin	mean	s.d.	Anx1 (P)	Anx5 (P)	Anx6 (P)	Nex1 (P)
None	0.5730	0.0171	0.00360	0.000070	0.000015	0.000013
Anx1	0.8653	0.0810		0.1065	0.00133	0.00138
Anx5	0.9717	0.0363			0.00068	0.00067
Anx6	1.2963	0.0468				0.8139
Nex1	1.2870	0.0441				

Figure 2d A600 at 1380 min

Annexin	mean	s.d.	Anx1 (P)	Anx5 (P)	Anx6 (P)	Nex1 (P)
None	0.8770	0.0200	0.00752	0.00181	0.000010	0.000012
Anx1	1.0240	0.0470		0.0101	0.000045	0.000043
Anx5	1.3250	0.1030			0.0133	0.00971
Anx6	1.5820	0.0200				0.2010
Nex1	1.610	0.0240				
